# One year of SARS-CoV-2 circulation in the Nouvelle-Aquitaine region, February 2021–2022, France

**DOI:** 10.3389/fmicb.2023.1176575

**Published:** 2023-07-28

**Authors:** Luc Deroche, Pantxika Bellecave, Romain David, Eric Ouattara, Magali Garcia, France Roblot, Laurence Boinot, Jean-François Faucher, Aurélie Rejasse, Guillaume Gschwind, Denis Malvy, Laurent Filleul, Sylvie Rogez, Nicolas Lévêque, Marie-Edith Lafon

**Affiliations:** ^1^Virology Laboratory, CHU Poitiers, Poitiers, France; ^2^Virology Laboratory, CHU Bordeaux, Bordeaux, France; ^3^Medical Information Analysis and Coordination Unit (UCAIM-DIM), Medical Information Department, Bordeaux University Hospital, Bordeaux, France; ^4^LITEC UR15560, Université de Poitiers, Poitiers, France; ^5^Tropical Infectious Diseases Department, Poitiers University Hospital, Poitiers, France; ^6^INSERM U1070, Université de Poitiers, Poitiers, France; ^7^Service d’Information Médicale, CHU Poitiers, Poitiers, France; ^8^Tropical Infectious Diseases Department, Limoges University Hospital, Limoges, France; ^9^Medical Information Department, Limoges University Hospital, Limoges, France; ^10^Department of Infectious and Tropical Diseases, CHU Bordeaux, Bordeaux, France; ^11^UMR Inserm 1219/IRD, University of Bordeaux, Bordeaux, France; ^12^Regional Office-Nouvelle Aquitaine, Santé publique France, Bordeaux, France; ^13^Virology Laboratory, CHU Limoges, Limoges, France; ^14^CNRS UMR 5234, University of Bordeaux, Bordeaux, France

**Keywords:** SARS-CoV-2, variant, epidemiological survey, Nouvelle-Aquitaine, France

## Abstract

**Background:**

Since 2021, 3 variants of concern (VOC) have spread to France, causing successive epidemic waves.

**Objectives:**

To describe the features of Alpha, Delta and Omicron VOC circulation in the Nouvelle-Aquitaine region, France, between February 2021 and February 2022.

**Study design:**

Data from the three university hospitals (UH) of Nouvelle-Aquitaine were used to describe regional SARS-CoV-2 circulation (RT-PCR positive rates and identified VOC) as well as its consequences (total number of hospitalizations and admissions in intensive care unit). They were analyzed according to the predominant variant and compared with national data.

**Results:**

A total of 611,106 SARS-CoV-2 RT-PCR tests were performed in the 3 Nouvelle-Aquitaine UH during the study period. The 37,750 positive samples were analyzed by variant-specific RT-PCR or whole-genome sequencing. In 2021, Alpha VOC was detected from week 5 until week 35. Delta became the most prevalent variant (77.3%) in week 26, reaching 100% in week 35. It was replaced by Omicron, which was initially detected week 48, represented 77% of positive samples in week 52 and was still predominant in February 2022. The RT-PCR positive rates were 4.3, 4.2, and 21.9% during the Alpha, Delta and Omicron waves, respectively. The ratio between intensive care unit admissions and total hospitalizations was lower during the Omicron wave than during the two previous waves due to the Alpha and Delta variants.

**Conclusion:**

This study highlighted the need for strong regional cooperation to achieve effective SARS-CoV-2 epidemiological surveillance, in close association with the public health authorities.

## Background

Since the first description of SARS-CoV-2-infected patients in France at the beginning of 2020 (Lescure et al., 2020; Stoecklin et al., 2020), up to nine successive epidemic waves, i.e. periods where the number of infected people increases to a peak and then declines—have passed over the country. In 2021, the observed peaks of infection were related to the emergence of new variants of concern (VOCs), Alpha variant from January to May, Delta from June to December and Omicron since December and until today.

The first description in France of the Alpha variant on December 13th 2020 led to the implementation of a national surveillance program to monitor the circulation of VOCs, either through PCR detection of specific mutations in Spike (S) protein (S:del69-70, S:N501Y, S:E484K/Q, S:L452R, S:K417N) or whole-genome sequencing (WGS; Bal et al., 2021; Gaymard et al., 2021). PCR detection of specific VOC mutations has been adapted throughout the epidemic, as variants have evolved. Flash surveys, during which a proportion of the SARS-CoV-2-positive samples on a given day were sequenced using WGS at regional and national levels, associated with the systematic screening for specific mutations of current VOCs, allowed monitoring of the circulation and emergence of SARS-CoV-2 variants (Bal et al., 2021; Gaymard et al., 2021) This genomic surveillance enabled the identification of clusters or imported cases, followed by the prevention of new variant spreading by reinforcing barrier measures and contact tracing (Anonymous, 2021; Hogan et al., 2021; Aggarwal et al., 2022).

From the beginning of the pandemic, the three university hospitals (UH) of the Nouvelle-Aquitaine region (Bordeaux, Limoges and Poitiers) decided to interact on a regular basis, in order to share technical problems, data and epidemiological evolution.

Objectives: The aims of the study are to report the COVID-19 epidemic features occurring in South-Western France between February 2021 (week 5/2021) and February 2022 (week 4/2022) by comparing (i) the kinetics of Alpha, Delta and Omicron variant appearance and (ii) their impact on hospitalization.

## Study design

### Study population

The Nouvelle-Aquitaine region counts over 6 million inhabitants and represents the third most populated French region (8.8% of French population). It has three UHs located in the cities of Bordeaux, Limoges and Poitiers. Bordeaux is located in the south of Nouvelle-Aquitaine and received respiratory samples from hospitalized patients as well as non-hospitalized patients (symptomatic people, contact tracing, mandatory PCR tests for travelling and Bordeaux international airport). Limoges, located at the East of the region, and working in conjunction with other geographically close hospitals, performed analysis mainly for hospitalized patients. Poitiers is a city in the North of the region. This laboratory analyzed samples from both hospitalized and non-hospitalized patients, working with a network of hospitals centers and private laboratories covering the north of the region.

### SARS-CoV-2 screening: PCR methods and tools of regional surveillance of SARS-CoV-2 variants

SARS-CoV-2 RNA was detected from respiratory samples by real-time RT-PCR (SARS-COV-2 R-Gene, Biomerieux, Craponne, France; AptimaTM SARS-CoV-2 assay, Panther system, Hologic, United States; Thermo Fisher Scientific TaqPath^™^ COVID-19 reverse-transcription polymerase chain reaction (RT-PCR) Kit, Thermo Fisher Scientific, Waltham, MA, United States). Variant-specific RT-PCR tests (VirSNiP assay, TIB Molbiol, Berlin, Germany or EurobioPlex SARS-CoV-2 SNPs, Eurobio Scientific, Les Ulis, France) were subsequently performed on SARS-CoV-2 positive samples. These tests were adapted according to epidemiological situations and governmental guidelines. First, tests detecting S:del69-70 and S:N501Y were carried out for Alpha surveillance in February 2021, quickly followed by S:E484K/Q (Beta and Gamma VOCs). After initial description of Delta VOC, PCR specific to S:L452R, (Delta) was developed from May 2021 whereas S:del69-70 and S:N501Y PCRs were discontinued. With the emergence of Omicron on December 2021, a PCR detecting S:K417N was added to screening; indeed, a combination of E484K/Q negative/ L452R negative and K417N positive PCR results led to the suspecting of an Omicron infection. In addition, whole genome sequencing of selected samples (i.e., unusual profile on screening, severe form of COVID-19, weekly flash surveys…) was performed using ARTIC-based protocols on Minion (Oxford Nanopore) or NextSeq550 (Illumina) sequencers followed by clade and Pango lineage determinations (Tyson et al., 2020; Aksamentov et al., 2021; O’Toole et al., 2021). Data were submitted to EMERGEN, the French genomic surveillance database, as well as GISAID.

### Hospitalized population

The number of hospitalized patients between week 5/2021 and week 4/2022 with a COVID-19 diagnosis, both total and in intensive care unit (ICU), was collected in each hospital.

## Results

### SARS-CoV-2 screening in Nouvelle-Aquitaine, February 2021: February 2022

A total of 611,106 respiratory samples were analyzed in the three UHs (53,063, 346,303 and 211,740 PCRs for Limoges, Poitiers and Bordeaux, respectively) of which 37,750 were positive for SARS-CoV-2. Positive samples were then subjected to variant-specific RT-PCR tests and whole-genome sequencing for variant monitoring.

### Circulation of VOCs

**Figure 1:**
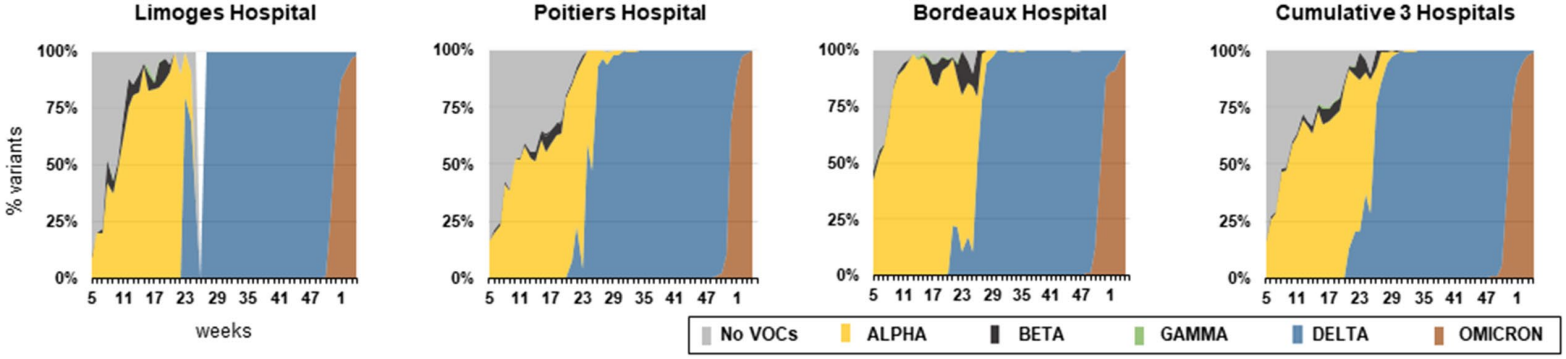
Percentage of VOCs detected for each hospital and cumulative data between W5/2021 and W4/2022. The VOCs were determined either by PCR detection of specific mutations harbored by VOCs (del69-70, N501Y, E484K/Q, L452R) or by sequencing. For Limoges Hospital, data were not available at Week 26.

The main characteristics of the 3 VOC waves observed between week 5/2021 and week 4/2022 are detailed in Table 1. The week of VOC first detection, the week the variant became dominant and the SARS-CoV-2 screening PCR positivity rate (peak and mean) were indicated for the 3 Nouvelle-Aquitaine UH and compared to national data.^1^ The percentage of the 5 VOCs (Alpha, Beta, Gamma, Delta, and Omicron) during this period is shown in Figure 1.

**Table 1:** Main characteristics of the 3 VOC waves observed between February 2021 (Week 5/2021) and February 2022 (Week 4/2022) in the 3 UHs of Nouvelle Aquitaine compared to national data.

		France	Nouvelle-Aquitaine		
			Bordeaux	Poitiers	Limoges
Alpha	First detection (Week)	W5	W5	W5	W5
	Dominant variant (Week, %)	W9 (68.2%)	W6 (53.0%)	W10 (52.5%)	W11 (60.2%)
	Highest positivity rate (%)	10.4	6.5	6.0	7.4
	Mean positivity rate (%)	6	4.1	4	5.6
Delta	First detection	W17	W20	W21	W23
	Dominant variant (Week, %)	W26 (42.9%)	W26 (50%)	W24 (59.5%)	W23 (80%)
	Highest positivity rate (%)	15.8	8.1	6.5	5.6
	Mean positivity rate (%)	6.8	4.6	3.4	3.4
Omicron	First detection	W46	W48	W49	W50
	Dominant variant (Week, %)	W52 (66.1%)	W51 (54.2%)	W52 (68.4%)	W52 (69.1%)
	Highest positivity rate (%)	34.5	26.6	30.9	27.8
	Mean positivity rate (%)	29.8	20.2	23.1	16.9

The 2021 week of VOC first detection, the 2021 week of VOC dominance and its prevalence (%), and the SARS-CoV-2 screening PCR positivity rate (peak and mean of percentage of SARS-CoV-2 positive PCRs) are listed.

### Alpha, Beta, and Gamma VOCs

Alpha was detected in the three UHs from week 5/2021 to week 35/2021 and represented 24.9% of all positive samples during this one-year study. Alpha established earlier in the Bordeaux area (week 6/2021) compared to both regional (week 11/2021 in Limoges and week 10/2021 in Poitiers) and national data (week 9/2021). It was the most prevalent variant in week 6/2021 (53.0%), 10/2021 (52.5%) and 11/2021 (60.2%) in Bordeaux, Poitiers and Limoges, respectively, and was predominant until week 25/2021. However, it never reached 100% of circulating strains (Table 1). Indeed, co-circulation of Beta and Gamma was observed between weeks 5–35/2021, which represented 1.2 and 0.1% of positive samples, respectively.

### Delta VOC

Delta was first detected in Bordeaux (week 20/2021) followed by Poitiers (week 21/2021) and Limoges (week 23/2021). Compared to national data (dominant variant at W26/2021), this VOC spread quickly in our region since it was the most prevalent in weeks 23/2021 and 24/2021 in Limoges (59.5% of variants) and Poitiers (80% of variants), respectively. In Bordeaux, Delta represented half of the SARS-CoV-2 positive-screening PCRs in week 26/2021. It was found in 100% of the samples examined from week 35/2021 to 46/2021, or, globally, in 29.5% of the samples examined during the study.

### Omicron VOC

Although first detection was reported in week 46 in France, our 3 UHs started identifying Omicron between week 48/2021 and 50/2021. In week 51/2021, it became the most prevalent circulating variant in Bordeaux (54.2%). In week 52/2021, it represented 68.4 and 69.1% of identified variants in Poitiers and Limoges, respectively. At the end of the study period (week 4/2022), its prevalence reached 99.4% in the three UHs. Altogether, Omicron was found in 26.9% of all positive samples of this study.

### Impact of each VOC on positivity rate of SARS-CoV-2 screening PCR

**Figure 2:**
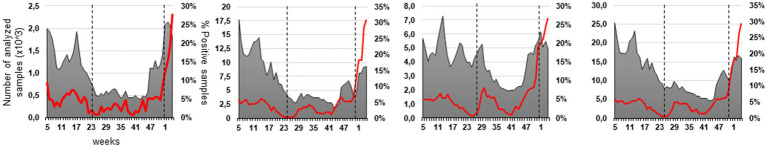
Number of PCR tests analyzed (grey) and percentage of SARS-CoV-2 positive PCRs (red) for each hospital and cumulative data between W5/2021 and W4/2022. The vertical dashed lanes separate the 3 VOCs-induced epidemics waves (i.e. when a VOC is dominant).

The total number of screening PCR tests and their percentage of positivity according to the waves induced by Alpha, Delta and Omicron were analyzed (Figure 2). The regional waves were defined as the period of VOC dominance (for cumulative regional data: Alpha: week 6/2021 to 25/2021; Delta: week 26/2021 to 51/2021; Omicron: week 52/2021 to week 4/2022). The mean percentage of positivity of screening PCR tests obtained by merging the 3 UH data (cumulative) were 4.3, 4.2, and 21.9% for Alpha, Delta and Omicron waves, respectively (Figure 2). These values were slightly lower than those observed at the national level, i.e., 6, 6.8, and 29.8%, respectively (Table 1).

### Impact of each VOC on hospitalizations

**Figure 3:**
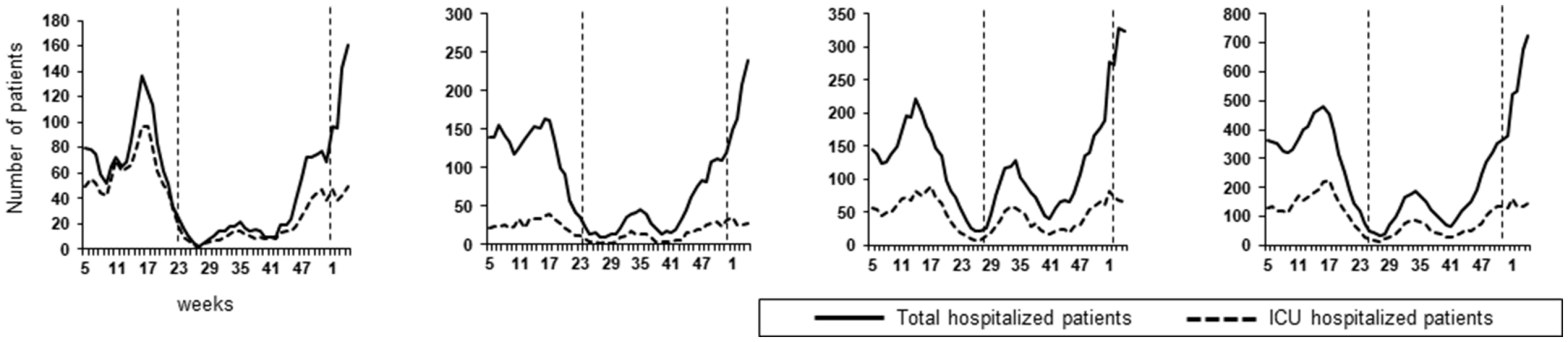
Total Number of hospitalized patients and ICU hospitalized patients between W5/2021 and W4/2022 for each hospital and cumulative data. The vertical dashed lanes separate the 3 VOCs induced epidemics waves (i.e. when a VOC is dominant).

Figure 3 presents the number of patients hospitalized with a COVID-19 diagnosis, including those in ICUs. The mean numbers of hospitalized patients per week in the 3 UH were 317, 153 and 568 during Alpha, Delta and Omicron waves, respectively. Nationwide, the mean numbers of hospitalized patients per week were 7,774, 3,474 and 15,761 during Alpha, Delta and Omicron waves (data not shown). In addition, mean numbers of ICU patients per week in the 3 UHs were 133, 61 and 142. The percentage of ICU hospitalization versus total hospitalized patients in the 3 UHs was similar between the Alpha (42%) and the Delta (41%) waves. These percentages were higher than those observed at the national level (22.4 and 23.1% for Alpha and Delta respectively). During the Omicron wave, the percentage of ICU hospitalized patients vs. total hospitalized patients was lower, with 26% for the 3 UHs versus 13.1% nationwide.

## Discussion

The cumulative data of the 3 UHs of the Nouvelle-Aquitaine region showed regional characteristics of the SARS-CoV-2 epidemic, which seemed to slightly differ from the national features.

Alpha was detected as soon as February 2021 in Nouvelle-Aquitaine (Figure 1). The early settlement of Alpha in Bordeaux area (week 6/2021) compared to other regional centers may have been due to the inclusion of SARS-CoV-2 tests from people travelling through Bordeaux airport, which may have led to multiple sources of Alpha importation. As previously described, the high transmissibility properties of Alpha led to an increase in positive screening PCR rates (Davies et al., 2021). However, the percentage of SARS-CoV-2 positive PCRs remained lower in our region compared to the national level. Indeed, our region has been relatively preserved from this wave, as had been the case with previous waves. A national lockdown was established from week 14/2021 to week 17/2021 to mitigate the Alpha wave, leading to a decrease in testing and, more importantly, a reduction in positive samples.

Beta and Gamma were also detected at low levels in the 3 UHs at the same period. These VOCs were detected in travelers or among clusters of infected people sharing a geographicarea in a given period of time, but their spreading was limited by the social confinement promoted by health authorities and viral genomic properties (founder effect). Notably, in May 2021, the Bordeaux laboratory first detected 5 patients infected with an Alpha variant carrying the E484Q mutation. This VOC was quickly detected in 102 cases corresponding to three main transmission chains in the Bordeaux metropolitan area. Preventive measures were rapidly initiated by the local regional health agency and Santé Publique France to contain this mutated VOC spreading (Evain S; manuscript in preparation).

The first Delta variants were described in Bordeaux UH, in May 2021, in patients having returned from India where this VOC initially emerged, in the context of industrial cooperation and university student exchange. Despite reinforced control by health authorities, Delta spread to the Nouvelle-Aquitaine region within 3 weeks, representing almost one third of sequenced SARS-CoV-2 in week 23/2021. Conversely, other French regions were slowly affected, since this variant represented only 9.3% in Paris and surroundings, and 8.5% in national data at the same week. This early detection was probably due to a cluster in a retirement home located in a southern department of the Nouvelle-Aquitaine region—leading to subsequent contamination of residents and caregivers followed by rapid dissemination. The three UHs were requested to divide up sequencing assays to quickly identify the Delta variant. Then, the replacement of Alpha by Delta within 1 month was in concordance with the United Kingdom epidemic features (Challen et al., 2021). During this period, the mean rate of SARS-CoV-2 positive screening PCR was slightly higher in Bordeaux compared to Limoges and Poitiers. We hypothesized that this higher percentage could be a local bias due to people who came for summer holidays to this highly touristic area. Surprisingly, despite the higher reproduction rate of the Delta, the total number of tests performed per week and the percentage of positive samples remained lower than during the Alpha wave (Dhar et al., 2021). Several factors may have contributed to these values: (i) the Delta wave settled during summer, a season which may have impaired viral transmission (Mandal et al., 2022); (ii) starting from the 23rd of May 2021, vaccination against SARS-CoV-2 was progressively extended to the general population; vaccination coverage with complete immunization was achieved for 36% of the Nouvelle-Aquitaine population at the start of July; (iii) restricted access to cultural or entertainment places to persons not immune to SARS-CoV-2, controlled by the delivery of a national “sanitary pass” (i.e., proof of vaccination or infection) or proof of a negative RT-PCR test within less than 48 h. This sanitary pass may have drastically reduced the number of screening tests, as more people were able to access places without restriction once vaccinated; and (iv) private medical laboratories progressively acquired PCR platforms, allowing them to perform SARS-CoV-2 RT-PCR without relying on their closest UH. Despite its low prevalence, circulation of Delta carried on until week 43/2021, with an increased number of positive PCRs suggesting the beginning of a new wave due to Delta in November 2021.

However, Omicron emerged in South Africa in November 2021 and spread worldwide within a few weeks (Paton et al., 2022). First detection in the Nouvelle-Aquitaine region occurred in weeks 48–49/2021. No major differences in regional and national data were observed during the start of the Omicron wave, as this VOC quickly replaced Delta in less than 4 weeks from e the day of first detection, as reported in other countries (Viana et al., 2022). In contrast to the previous waves, Omicron emergence affected Nouvelle-Aquitaine with the same intensity as in other French regions. The high rate of screening PCR positivity could be explained by the enhanced transmissibility of Omicron and the decreased protective effect of vaccinal strategies associated with omicron’s increased immune system escape (Viana et al., 2022).

Interestingly, detection of both Delta and Omicron sequences occurred in 35 samples during the Omicron wave in Poitiers. Analysis of sequences suggested coinfection with the two variants, rather than a recombinant virus. Coinfection, as well as recombinant virus, have been noted, but they remain extremely rare relative to the huge number of infections (Bal et al., 2022; Combes et al., 2022; Rockett et al., 2022).

Finally, we analyzed the impact of the VOCs on hospitalizations. The total absolute number of hospitalizations was higher during the Omicron wave, followed by the Alpha and then the Delta waves, similarly to national data. However, the ratio of hospitalized vs. PCR-positive patients during the Omicron period was lower. We were not able to calculate the exact rate of hospitalized patients compared to the number of positive PCR tests, because long-term hospitalized patients were sampled several times. The ratio of ICU hospitalized patients vs. total hospitalized patients was similar between the Alpha and the Delta waves in the 3 UHs; it was lower during the Omicron wave due to its lesser severity (Madhi et al., 2022). It has been mentioned that patients infected with the Delta had a higher risk of hospital admission in comparison with patients infected with the Alpha (Twohig et al., 2022). This was not observed in our hospitals. This difference might be due to the vaccination status of the study population. Indeed, in a United Kingdom study, almost 75% of the infected patients were unvaccinated or with an incomplete vaccination scheme. We could assume that in Nouvelle-Aquitaine region, the level of complete vaccination (36% at the beginning of July 2021 and 78% on November 2021) offered an effective protection against severe forms of COVID-19 induced by Delta. Unfortunately, vaccination status was not available for hospitalized patients.

## Conclusion

All in all, this regional descriptive study showed the impact of SARS-CoV-2 variability on COVID-19 epidemics over a one-year period, during which 3 different variants—with increased rate of transmissibility and different disease severity—emerged. Efforts on genomic surveillance should be carried on to detect new SARS-CoV-2 variants. Our organizational capacity allowed us to set up efficient regional epidemiological surveillance, which provided public health authorities with useful decision tools to monitor epidemics at the local and national levels. Such an organization is necessary and additional to other surveillance tools such as detection of SARS-CoV2 variants in sewage effluents. The valuable regional data shared by the three Nouvelle Aquitaine UHs underlines, for the future, the interest of strong regional cooperation in such a long-lasting epidemic crisis.

## Data availability statement

The original contributions presented in the study are included in the article/supplementary material, further inquiries can be directed to the corresponding author.

## Ethics statement

Ethical review and approval was not required for the study on human participants in accordance with the local legislation and institutional requirements. Written informed consent for participation was not required for this study in accordance with the national legislation and the institutional requirements.

## Author contributions

SR, NL, and M-EL coordinated the study. RD, EO, LB, AR, and GG collected the data. PB and LD analyzed the data with the support from RD. PB, LD, SR, NL, and M-EL interpreted the results and wrote the manuscript. DM, LF, MG, FR, and J-FF provided critical review of the text. All authors contributed to the article and approved the submitted version.

## Funding

Funding was provided from the three university hospitals, the Agence Régionale de Santé of Nouvelle-Aquitaine and the Region Nouvelle-Aquitaine Council.

## Conflict of interest

The authors declare that the research was conducted in the absence of any commercial or financial relationships that could be construed as a potential conflict of interest.

## Publisher’s note

All claims expressed in this article are solely those of the authors and do not necessarily represent those of their affiliated organizations, or those of the publisher, the editors and the reviewers. Any product that may be evaluated in this article, or claim that may be made by its manufacturer, is not guaranteed or endorsed by the publisher.

## Footnotes

^1^https://geodes.santepubliquefrance.fr
